# Molecular and cellular dynamics of squamous cell carcinomas across tissues

**DOI:** 10.1101/gad.351990.124

**Published:** 2025-01-01

**Authors:** Matthew R. Kudelka, Yonit Lavin, Siman Sun, Elaine Fuchs

**Affiliations:** 1 Howard Hughes Medical Institute, Robin Chemers Neustein Laboratory of Mammalian Cell Biology and Development, The Rockefeller University, New York, New York 10065, USA;; 2 Department of Medicine, Memorial Sloan Kettering Cancer Center, New York, New York 10065, USA

**Keywords:** cancer epigenetics, cancer genetics, cancer signaling, cancer stem cells, epithelial cancers, squamous cell carcinoma, translational regulation, tumor microenvironment

## Abstract

Squamous cell carcinomas (SCCs), arising from the skin, head and neck, lungs, esophagus, and cervix, are collectively among the most common cancers and a frequent cause of cancer morbidity and mortality. Despite distinct stratified epithelial tissues of origin, converging evidence points toward shared biologic pathways across SCCs. With recent breakthroughs in molecular technologies have come novel SCC treatment paradigms, including immunotherapies and targeted therapy. This review compares commonalities and differences across SCCs from different anatomical sites, including risk factors and genetics, as well as cellular and molecular programs driving tumorigenesis. We review landmark discoveries of the “cancer stem cells” (CSCs) that initiate and propagate SCCs and their gene and translational regulation programs. This has led to an appreciation that interactions between CSCs and the immune system play key roles in invasion and therapeutic resistance. Here, we review the unifying principles of SCCs that have emerged from these exciting advances in our understanding of these epithelial cancers.

## Abstract (precis)

In this review, Kudelka et al. examine the unifying cellular and molecular principles that contribute to the development and progression of squamous cell carcinomas (SCCs). They discuss cell-autonomous features, like genetics and cancer-initiating stem cells, and non-cell-autonomous features, like the immune microenvironment, that contribute to this cancer despite being in different anatomical sites.

Stratified epithelial tissues form the body's barrier to the outside world. As our climate rapidly changes, these barrier epithelial tissues are subjected to a myriad of external assaults, including an increasing diversity of air pollutants, pollens and allergens, and pathogens. As our ozone layer thins, harmful UV radiation adds to this array of potentially carcinogenic onslaughts (Hyde 1906). Such stimuli often elicit inflammation, which appears to be a unifying instigator in elevating cancer risk (Dolberg et al. 1985; Greten and Grivennikov 2019).

To shield the body from attacks, normal stratified epithelia maintain a population of adult stem cells, which reside along a basement membrane, rich in collagen IV, other extracellular matrix (ECM) proteins, and growth factors, enabling stem cells to both self-renew and give rise to protective layers of differentiating cells at the body's interface with the environment (Tumbar et al. 2004; Lechler and Fuchs 2005). The high proliferative capacity of healthy stratified tissues places them at increased risk of cancer (Tomasetti and Vogelstein 2015), and when coupled with the assaults from the external environment, susceptibility rises further (Pott 1775). Thus, squamous cell carcinomas (SCCs) are typified by a high mutational burden and chromosomal instability, which can activate RAS/MAPK and other pathways, further fueling proliferation (Campbell et al. 2018) and ultimately driving breakdown of the basement membrane and invasion into underlying stroma.

Although screenings and awareness have had a major impact on SCC-related deaths, particularly for cervical, cutaneous, and smoking-related lung SCCs, advanced disease remains challenging to treat (Paz-Ares et al. 2018; Burtness et al. 2019; Grob et al. 2020). Grouped together, SCCs account for nearly 2 million, or one-fifth, of cancer deaths each year worldwide (Bray et al. 2024). Until recently, treatments were limited to surgical resection and radiation and/or platinum-based therapies. Advances in immune and targeted therapies now extend survival beyond chemotherapy-based approaches; however, patients with metastatic SCCs still succumb to their disease (Migden et al. 2018; Paz-Ares et al. 2018; Burtness et al. 2019; Colombo et al. 2021).

To develop new and improved therapies, it is critical to understand the oncogenic mutations that normal epithelial stem cells acquire and the ensuing malfunction in communication between the oncogenic stem cells and their microenvironment, as together, these genetic and epigenetic facets ultimately lead to malignant, invasive, and metastatic SCCs. Emerging basic, translational, and clinical studies point to a common biology of SCCs. In this review, we explore the common genetic mutations and key molecular pathways that contribute to tumorigenesis in SCCs. We specifically delve into the role of a stem cell-like compartment in SCCs and the significance of immune responses of SCCs across body sites. By moving away from tissue specificity toward the similarities of SCCs across body sites, new insights are beginning to emerge with the promise of driving more rapid advances in treatment of these diseases.

## Key mutational pathways in squamous cell carcinomas

SCCs can arise from exposure to carcinogens, which eventually give rise to oncogenic mutations that drive pathogenesis (Khuder 2001; Mork et al. 2001; Morita et al. 2010; Schmitt et al. 2011; Que et al. 2018; Lei et al. 2020; Mody et al. 2021). Many similar gene mutations are found in SCCs across tissue sites, which have given researchers critical insights into key factors and signaling pathways necessary for the pathogenic switch to invasion (Campbell et al. 2018; Sánchez-Danés and Blanpain 2018). Although *HRAS* is commonly hyperactivated in cutaneous and head and neck SCCs, *KRAS* mutations are more often seen in lung and cervical SCCs.

**Figure 1.**
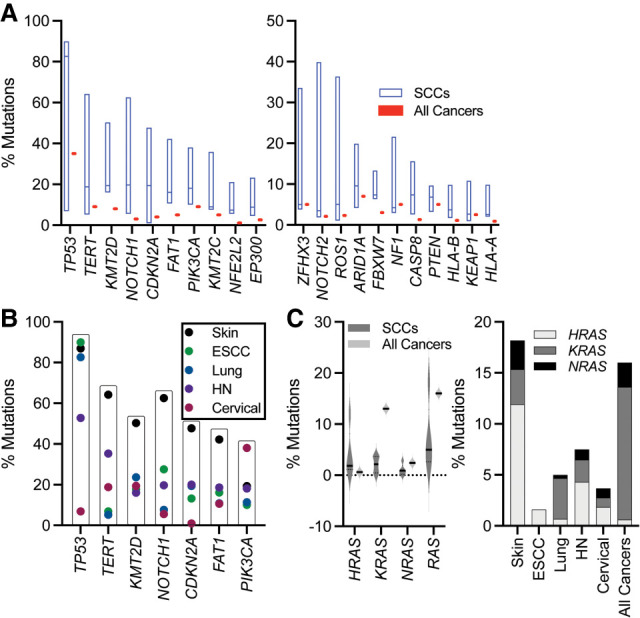
Common mutations in human SCCs versus all cancers. (*A*) Frequency of mutated genes per each SCC (skin, ESCC, lung, head and neck [HN], and cervical) as compared with all cancers. (*Left*) Frequent SCC mutations. (*Right*) Less frequent but still appreciable mutations in SCCs. Median is denoted with a line. Bars indicate range. (*B*) Most frequently mutated genes across SCCs. Note the low frequency of *TP53* mutations in cervical SCC, explained by the high incidence of HPV, where TP53 is inactivated by the E6 viral oncoprotein. Additionally, there is a low frequency of *CDKN2A* mutations in this cancer, corresponding to a high frequency of RB inactivation by the E7 viral oncoprotein. (*C*) Mutations in *RAS*. (*Left*) A violin plot with the frequency of all SCCs plotted versus all cancers grouped together. (*Right*) Frequency of different *RAS* genes in each SCC versus all cancers. (*A*–*C*) Mutation data from Memorial Sloan Kettering Integrated Mutation Profiling of Actionable Cancer Targets (MSK-IMPACT). *n*_samples_ = 176 skin SCC, *n*_samples_ = 189 esophagus (ESCC), *n*_samples_ = 1482 lung SCC, *n*_samples_ = 788 head and neck (HN SCC), *n*_samples_ = 218 cervical SCC, *n*_samples_ = 84,225 samples from 72,067 patients (all cancers). Note that all mutations are included. This overestimates driver mutations in UV-induced high TMB cSCCs. For example, if variants of unknown significance (VUSs) are excluded, *HRAS* mutations are present in 7% rather than 11% of samples. In a more extreme case, *ROS1* is mutated in 37% of cSCCs, but <1% have *ROS1* driver mutations. Similarly, 5% *KRAS* mutation in lung SCC is higher than expected. About 10% of these are VUSs. Also, pathology is based on a single biopsy, which could result in contamination of adenosquamous cancer into the lung SCC cohort, further overestimating the frequency of *KRAS* mutations.

**Figure 2.**
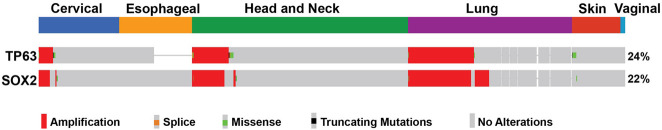
*TP63* and *SOX2* amplifications in human SCCs. Data were compiled from publicly available TCGA data on cBioPortal squamous cell carcinoma samples (Cerami et al. 2012; Gao et al. 2013). *n*_samples_ = 1833 as follows: *n*_samples_ = 297 cervical, *n*_samples_ = 227 esophagus, *n*_samples_ = 632 head and neck, *n*_samples_ = 511 lung, *n*_samples_ = 151 skin, *n*_samples_ = 15 vaginal.

In addition to *RAS*, activating mutations in *TERT* and *PIK3CA* are frequent across human SCCs (or upstream receptors that lead to RAS signaling, such as FGFR1 amplification) (Fig. 1; The Cancer Genome Atlas Research Network 2012; Campbell et al. 2018). Gene amplifications leading to elevated levels of key lineage-defining transcription factors such as TP63 and SOX2 have also been reported (Fig. 2), as have loss of function and/or dominant-negative mutations in *TP53*, *KMT2D*, *NOTCH1*, *CDKN2A*, and *FAT1* (Hippo signaling) (Martin et al. 2018; Pastushenko et al. 2021), which are among the most highly mutated genes shared across many albeit not all SCCs (Fig. 1A,B). *NOTCH* and *TP53* mutations, for instance, are more than five times more frequent in cutaneous SCCs than in cervical SCCs, whereas *TP63* mutations are more frequent in cervical than in esophageal or vulval SCCs. Even more perplexingly, *SOX2* amplifications are common in lung SCCs but rare if at all in cutaneous SCCs, and yet loss of *Sox2* in mice is critical for both cutaneous and lung SCCs (Boumahdi et al. 2014; Mukhopadhyay et al. 2014).

Whether such site-specific SCC differences are rooted in the specific carcinogens involved or in the tissue-specific tumor microenvironments of these cancers is a fascinating question, the answers to which are largely unknown. We revisit some of these points later in considering both the mutational burden and the molecular pathways that are common across SCCs, as reclassifying SCCs by common biological processes across tissues could reinvigorate a search for novel therapies.

### Stereotypic mutations from carcinogens

One of the primary risk factors for cSCC is sun exposure to ultraviolet (UV) light, primarily UV-B causing direct DNA damage but also indirect DNA damage through UV-A. This leads to stereotypic mutations with a signature of C>T transversions developed through pyrimidine dimer formation (Alexandrov et al. 2013). Smoking-related SCCs in the lungs, head and neck, and esophagus also have a stereotypic signature with G>T/C>A mutations from tobacco-associated carcinogens (Alexandrov et al. 2016). Finally, human papilloma virus 16/18 (HPV16/18) has been associated with a subset (oropharyngeal) of head and neck cancers (HNSCCs) and most cervical SCCs. Following integration of the virus into the host genome, expression of the HPV oncoproteins E6 and E7 results in the inactivation of host tumor suppressors TP53 and retinoblastoma RB protein, respectively. Given the importance of TP53 in the genotoxic damage repair pathway, HPV infections in cervical SCCs are likely to achieve what *TP53* gene mutations do in other SCCs, leading to host genetic instability and a high mutational burden.

The mutations generated by a wide variety of carcinogens to induce tumorigenesis eventually require activation or inactivation of prototypic oncogenes and tumor suppressors, respectively (Fig. 1). The high mutational burden induced by these carcinogenic insults may also increase the potential to generate neoantigens for T immune lymphocytes to recognize and concomitantly drive tumor acquisition of immune evasive strategies. Together, these intrinsic and extrinsic changes lead to tumorigenesis and tumor invasion.

The features of SCCs in humans are modeled in a variety of animal models that often require both carcinogenic exposure and ongoing insults. One of the most common mouse models for cSCCs is the topical application to mouse skin of the chemical mutagen dimethylbenz[a]anthracene (DMBA), which when followed by repetitive treatments of the tumor promoter 12-O-tetradecanoylphorbol-13-acetate (TPA) initiates benign tumors (papillomas) that progress to cSCCs. DMBA induces an A>T stereotypic mutational signature, and similar to human cSCCs, these cancers are characterized by recurrent mutations in *Hras* and, to a lesser extent, *Kras* and *Rras2* (Bizub et al. 1986; Quintanilla et al. 1986; Nassar et al. 2015).

A similar progression from dysplasia to carcinoma has been observed for other SCCs, such as adding the tobacco surrogate 4-nitroquinoline 1-oxide in drinking water to drive HNSCC and adding the mutagen diethylnitrosamine (DEN) to drive esophageal tumors (Tang et al. 2013; Frede et al. 2016; Chen et al. 2017; Colom et al. 2021). These models allow for exploring the complex roles of increasing mutational burden in cells within epithelial tissues under conditions where the tissue remains visibly normal versus the stage at which tumors begin to appear.

Like their chemically induced counterparts, genetically engineered mouse models of *Hras*^*G12V*^ or *Kras*^*G12D*^ can also initiate tumorigenesis (Caulin et al. 2004; Chen et al. 2009) and recapitulate the progression to invasive SCC (Yuan et al. 2022). In the presence of a second oncogenic hit or loss of a tumor suppressor such as *Tp53*, tumor progression is accelerated, with accompanying genetic instability reflected in copy number variations and aneuploidy (Ruggeri et al. 1991; Kemp et al. 1993; Lazarov et al. 2002; Dajee et al. 2003; Chen et al. 2009; Lapouge et al. 2011; White et al. 2011; Nassar et al. 2015).

Mouse SCC models, both mutagenic and transgenic, have recapitulated many of the key pathways and features of human SCC, and this has greatly facilitated the identification and characterization of the cell types (such as oncogenic stem cells) that initiate these tumors and the key pathways that drive their progression. As epigenetic modifiers, translational machinery, and the microenvironment are increasingly appreciated as key drivers of tumorigenesis, an in-depth understanding of the subtle differences between these models will be of paramount importance in developing new therapeutic avenues.

### TP53 and clonality

With *TP53* being mutated or suppressed (e.g., by HPV) in the majority of invasive, aggressive SCCs, it is surprising that TP53 loss and a subsequent high mutational burden can arise in the stem cells of the skin, lungs, and esophageal epithelium even when the tissue is aphenotypic (Nelson et al. 1996; Martincorena et al. 2015, 2018). These findings underscore the importance of the surrounding microenvironment in suppressing tumorigenesis.

One possible explanation might be rooted in cell competition, where more fit neighboring stem cells battle with less fit ones for a limited space within the basal progenitor layer (Murai et al. 2018). As mutations accumulate, a cell that is more fit and expanding within the tissue can suddenly become less fit and eliminated from the tissue. In this way, cell competition might preserve tissue fitness in the face of mutational burden until a stem cell has not only acquired a survival advantage but also found itself among loser neighbors, creating a perfect storm to progress to a cancerous lesion (Martincorena et al. 2015; Colom et al. 2021).

Support for this model comes from fate-mapping studies in the skin of mice that harbor a “Brainbow” cassette, composed of one promoter and a diverse array of individually floxed encoded fluorescent proteins. When tamoxifen is administered at various time points during tumorigenesis, genomic rearrangement occurs such that each stem cell expresses and propagates one fluorescent marker to its progeny. Analyses of the ensuing fluorescent clones revealed that in benign papilloma, there are dominant clones sustained by cancer stem cells, similar to intestinal adenoma (Schepers et al. 2012; Nicholson et al. 2018), but during tumor progression, there is a clonal selection during which only one clone is responsible for malignant transition (Driessens et al. 2012; Reeves et al. 2018).

Interestingly, in some settings, skin is resistant to mutations in some canonical cancer genes (Ying et al. 2018). Although these resistance mechanisms are not universal across tissues or in all scenarios in skin (Van Keymeulen et al. 2015; Chang and Shain 2021), they highlight the importance of understanding how cell competition, cancer genes, and tissue biology interact to drive tumorigenesis. Although a deeper understanding of the molecular underpinnings of cell competition and clonal expansion is now needed, these findings underscore the importance of both dominant mutations and the microenvironment in governing the transition to malignancy and invasion.

### *TERT*, *PIK3CA*, *MMP*s, *KMT2D*, and *CDKN2A*

In SCCs, *TERT*, *PIK3CA*, and *matrix metalloproteinases* (*MMP*s) are common oncogenes, whereas *KMT2D* and *CDKN2A* are common tumor suppressors (Fig. 1A,B). Although these genes have been reviewed extensively elsewhere, we highlight salient features in SCCs. In terms of oncogenes, *TERT* promoter mutations are frequent in SCCs (Fig. 1A,B; Scott et al. 2014), resulting in increased telomerase activity, extended telomeres and additional functions (Stewart et al. 2002). Phosphoinositide 3-kinases (PI3Ks) comprise four classes of receptors. Class I is heterodimeric with regulatory and catalytic subunits. It activates AKT and is negatively regulated by PTEN. The p110a regulatory isoform encoded by *PIK3CA* is often mutated in human SCCs and is functionally important, as shown in mice (Fig. 1A,B; Loganathan et al. 2020). However, wild-type PI3K can also be hyperactivated, for example, by leptin receptor, through interaction with the tumor microenvironment, and this too can drive SCCs (Loganathan et al. 2020; Yuan et al. 2022). Analogously, *MMP* gene amplifications are common in ∼15% of SCCs (or ∼5% individually for *MMP1*, *MMP3*, *MMP7*, *MMP8*, *MMP10*, *MMP12*, *MMP13*, *MMP20*, and *MMP27*) (TCGA data via cBioPortal) and correlate with invasion (Sato et al. 1994; Wright et al. 1994; Wirtz et al. 2011). However, mechanical forces from the tumor itself and the tumor microenvironment can also markedly affect basement membrane breakdown (Fiore et al. 2020). Thus, as we discuss further below, SCCs, like most cancers, are not simply genetic disorders, but ones in which cross-talk between the cancer and its microenvironment can be instrumental drivers.

In terms of tumor suppressors, KMT2D is an epigenetic regulator required for monomethylation of H3K4 at distal regulatory enhancers. It is frequently mutated and can be a tumor promoter in some contexts, though its action appears to be tumor-suppressing in SCCs (Pan et al. 2023). *CDKN2A* is commonly inactivated in SCCs (Kamb et al. 1994; Soufir et al. 1999; Xing et al. 1999). In its wild-type state, *CDKN2A* encodes two tumor suppressors: p16/Ink4a inhibits cell cycle kinases CDK4/6 and keeps cells arrested through RB, whereas p14ARF (p19ARF in mice) activates TP53 (Serrano et al. 1993; Bates et al. 1998). Accordingly, head and neck and cervical SCCs with downstream inactivation of RB by HPV E7 are mutually exclusive for *CDKN2A* mutations, and interestingly, HNSCCs display feedback activation of p16, which is used clinically as a surrogate to identify HPV infection and a favorable prognosis (Fig. 1B; Lewis et al. 2010; Chung et al. 2014).

### Ras pathway as a key driver of SCCs

*RAS* genes, such as *HRAS*, *KRAS*, and *NRAS*, were the first identified human oncogenes and are mutated frequently in cancer (Malumbres and Barbacid 2003). *RAS* was discovered in rat sarcoma viruses in the 1960s (Harvey 1964; Kirsten and Mayer 1967) and subsequently in human cancer cells in the 1980s (Perucho et al. 1981; Shih et al. 1981; Der et al. 1982; Goldfarb et al. 1982; Parada et al. 1982; Pulciani et al. 1982; Santos et al. 1982; Shih and Weinberg 1982). When mitogens bind receptor tyrosine kinases (RTKs), they induce dimerization and autophosphorylation and trigger a cascade of signaling events that often begin with the recruitment of the guanine nucleotide exchange factor SOS and the subsequent activation of RAS-GTP. This in turn activates RAF and mitogen-activated protein kinase (MAPK), which then phosphorylates and activates the nuclear extracellular signaling-regulated kinase (ERK), a regulator of transcription factors that control cell growth, proliferation, and survival. Oncogenic *RAS* mutations maintain a GTP-bound active conformation, rendering the MAPK pathway constitutive, as reviewed elsewhere (Lemmon and Schlessinger 2010; Jeon et al. 2024)

About 10% of SCCs have activating mutations in *RAS* (Fig. 1C). SCCs of the skin and head and neck are particularly enriched for *HRAS* mutations, whereas most other SCCs are enriched for *KRAS* mutations. Of the non-SCCs harboring *RAS* mutations, *KRAS* (adenocarcinomas of the pancreas and lung) > *NRAS* (melanomas) > *HRAS* (salivary duct carcinoma) (Cerami et al. 2012; Gao et al. 2013; Lin et al. 2014; Pickering et al. 2014; Song et al. 2014; Li et al. 2015; Sanchez-Vega et al. 2018; Chang and Shain 2021). With the exception of melanoma, *RAS* mutations in nonepithelial cancers tend to be rare (Zehir et al. 2017).

Given the prevalence of *RAS* mutations in SCCs, it is not surprising to see that RTK–RAS–MAPK signaling is a major driver of tumorigenesis. Both DMBA/TPA and genetically engineered mouse models of *Hras*^*G12V*^ or *Kras*^*G12D*^ can drive tumorigenesis, as reviewed elsewhere (Yuan et al. 2024). Although TP53 loss greatly accelerates oncogenic progression (Baslan et al. 2022), elevation of RAS expression levels alone through *Ras* amplification (Quintanilla et al. 1986) or transgene expression can induce an epithelial stem cell to reprogram its microenvironment and, through continued aberrant cross-talk, leads to a malignant, invasive phenotype characteristic of SCCs (Beronja et al. 2013; Oshimori et al. 2015; Yuan et al. 2022).

Strong links between RAS/MAPK and human SCCs are also well established. Thus, for instance, among the RASopathies (a cohort of genetic disorders in humans whose tissues are overactivated for the RAS/MAPK pathway), Costello syndrome is caused by heterozygous activating mutations in *HRAS* and is associated with a high incidence of skin papillomas (Rauen 2013). A somewhat paradoxical link comes from melanoma patients placed on BRAF inhibitors, who subsequently develop cSCCs resulting from the superactivation of MAPK in skin epithelial cells that are wild type for *BRAF* (Flaherty et al. 2012; Oberholzer et al. 2012; Su et al. 2012).

Although historically undruggable, RAS inhibitors recently entered the clinic, including FDA-approved *KRAS*^*G12C*^ inhibitors (Skoulidis et al. 2021; Jänne et al. 2022) and investigational inhibitors, such as tipifarnib for *HRAS* mutant HNSCCs (Ho et al. 2021). Upstream, cetuximab blocks EGFR and is FDA-approved for cSCCs (Maubec et al. 2011) and HNSCCs (Vermorken et al. 2008).

### NOTCH

NOTCH1–4 are transmembrane proteins whose activation is dependent on a neighboring cell presenting sufficient levels of its transmembrane ligands (JAG1/2 or DLL1/3/4). Following receptor–ligand engagement, the NOTCH extracellular domain is cleaved by a protease, triggering γ-secretase-mediated cleavage of the NOTCH intracellular domain (NICD). Freed of its transmembrane association, NICD translocates to the nucleus, where it interacts with DNA binding partner RBPJ to activate transcription of downstream target genes, such as *Hes* and *Hey* (Blanpain et al. 2006; Nowell and Radtke 2017).

In the skin epidermis, *NOTCH* plays a critical role in driving epidermal differentiation and suppressing basal cell fates (Blanpain et al. 2006; Watt et al. 2008; Zanconato et al. 2015). *NOTCH1–4* are frequently mutated in skin SCCs and HNSCCs (Agrawal et al. 2011; Stransky et al. 2011; Wang et al. 2011; Gao et al. 2014; Pickering et al. 2014; Rampias et al. 2014; Song et al. 2014; South et al. 2014), where they function as tumor suppressors (Nicolas et al. 2003; Nowell and Radtke 2017). For example, *Notch1* deletion results in spontaneous tumors or accelerated tumorigenesis following a mutagen (DMBA) and inflammatory insult (TPA) (Nicolas et al. 2003). This is in part because *NOTCH* mutations lead to aberrant immune signaling and inflammation in the skin, contributing to tumorigenesis (Demehri et al. 2008, 2009, 2012; Dumortier et al. 2010; Di Piazza et al. 2012; Murthy et al. 2012), as observed in other models (Perez-Moreno et al. 2008).

In some cancers, *NOTCH* mutations can be tumor-promoting (Ferrando 2009), indicating that context and tissue type can impact the outcome of these mutations in cancer. In this regard, it is intriguing that *NOTCH1* mutations are much less frequent in esophageal SCCs relative to those of skin or head and neck and yet can accumulate in the aphenotypic adult esophagus (Abby et al. 2023). Although still speculative, the ability of *NOTCH* mutations to drive clonal expansion in normal esophagus is likely to be rooted in yet another example of cell competition played out at clonal boundaries. Thus, to maintain homeostasis, *NOTCH* wild-type cells adjacent to mutant neighbors must devote extra time toward differentiation at the expense of basal expansion. This enables *NOTCH* mutant basal cells to clonally expand even if they divide less efficiently than their wild-type counterparts (Abby et al. 2023).

### Transcription factor amplification

One interesting feature of SCCs is that they frequently have amplifications or hyperactivation of key lineage-defining transcription factors. One key transcription factor is TP63, which is expressed normally by all epithelial stem cells in steady state (Truong et al. 2006). In SCCs, most mutations in *TP63* appear to be gain of function or amplifications. Genetic studies in the skin of mice point to the view that the transactivating “TA” form of TP63 suppresses SCC progression, whereas the alternatively spliced ΔNTP63 form, the one expressed typically in basal epithelial stem cells, causes hyperplasia and enhanced susceptibility to SCCs when deregulated (Devos et al. 2017). In the lung, the ΔNTP63 isoform is used as a biomarker to distinguish lung SCCs from adenocarcinomas (Bishop et al. 2012). In part, ΔNTP63's action could be rooted in its ability to interfere with TP53 function, though it has also been implicated as antagonistic to NOTCH signaling. Both of these roles would favor tumor progression.

An additional twist is that in lung, head and neck, esophageal, and cervical SCCs, the 3q chromosomal region is often amplified, and this region contains not only *TP63* but also *SOX2*, which encodes another transcription factor widely involved in tumor progression among SCCs from different body sites (Fig. 2). In contrast to internal stratified tissues such as the lung airway, SOX2 is not normally expressed in skin epidermis. Although its gene is not amplified in cSCCs, SOX2 is highly expressed in both mouse and human cSCCs as well as in human actinic keratosis, a premalignant condition (Schober and Fuchs 2011). Notably, *Sox2* ablation in mice causes tumors to regress (Boumahdi et al. 2014), and in mouse lungs, *Sox2* overexpression drives SCC histology not only in SOX2^+^ airway epithelia but also in alveolar tissue, which, like epidermis, does not normally express SOX2 (Ferone et al. 2016). Collectively, these findings further underscore the importance of SOX2 upregulation in SCCs, whether by gene amplification or deregulation.

Given the importance of SOX2 to SCC formation, it still remains unclear how SOX2 functions and how it is regulated. On the one hand, SOX2 has been reported to inhibit NOTCH signaling (Xu et al. 2014b), and if so, it might be interesting to explore a possible synergistic action with ΔNTP63 (Watanabe et al. 2014). On the other hand, SOX2 may also affect the tumor microenvironment, as it has been reported to regulate neutrophil recruitment via chemokines and metabolism by enhanced expression of the glucose transporter GLUT1 in SCCs (Mollaoglu et al. 2018; Hsieh et al. 2019). Sifting through these possibilities may be complicated by the fact that in many SCCs, another family member, SOX9, is coexpressed, and it too appears to be essential for SCC maintenance (Ge et al. 2017). This will continue to be an important area of future research as its implications for therapeutics are considered.

## Cancer stem cells in squamous cell carcinomas

### History of cancer stem cells

The cancer stem cell (CSC) theory states that there is at least one cell within a tumor that can both regenerate itself (self-renew) and produce heterogeneous tumor progeny (Clarke et al. 2006). Researchers soon speculated that such a population might be responsible for relapse following cancer therapeutics, and that identifying, characterizing, and targeting CSCs could thus hold the key to a cure.

In 1875, Julius Conheim proposed a preluder to the CSC model: the “embryonal rest theory” (Cohnheim 1875), which states that adult tissues contain embryonic tissue that can be activated to become cancer. Teratocarcinoma and leukemia provided early evidence for CSCs. In the 1960s, Barry Pierce observed that teratocarcinomas consist of undifferentiated and differentiated cells with different mitotic properties (Pierce et al. 1960). He proposed that undifferentiated cells give rise to differentiated cells (Pierce et al. 1960), and in 1964, he performed single-cell transplants to show that embryonal carcinoma cells derived from trypsinization of small embryoid bodies enriched for these cells generated teratocarcinomas that contained both cell populations (Kleinsmith and Pierce 1964). Extending this notion further, James Till and Ernest McMcCulloch (Till and McCulloch 1961; Becker et al. 1963; Siminovitch et al. 1963) identified stem cells that could reconstitute the hematopoietic system upon limiting dilution transplantation (Hewitt and Wilson 1959), and this then inspired John Dick (Lapidot et al. 1994; Bonnet and Dick 1997) to test the CSC model in the 1990s. Dick's group (Lapidot et al. 1994; Bonnet and Dick 1997) discovered that a rare population of CD34^+^CD38^−^ cells in patient blood was able to initiate leukemia, including all differentiated lineages, upon transplantation into immunodeficient mice. These cells were able to self-renew and generate the entire leukemia hierarchy, providing the first formal evidence of CSCs.

Since its codification in the 1990s, the CSC model has been found to apply to many tumors (Lapidot et al. 1994; Bonnet and Dick 1997; Al-Hajj et al. 2003; Singh et al. 2004). CSCs need not be rare, and indeed, for melanomas, nearly all cells have the capacity to self-renew and propagate the cancer (Quintana et al. 2008, 2010). However, in certain tumors, such as SCCs, a subset of cells plays a key role in self-renewal, treatment resistance, and tumor propagation (Schober and Fuchs 2011; Lapouge et al. 2012).

### The existence of SCC cancer stem cells and an SCC cancer stem cell niche

The first hint of SCC CSCs came in 1971, when Pierce and Wallace (1971) labeled rats harboring cSCCs with tritiated thymidine and then traced this long-lived nucleotide over time. After observing initial labeling of undifferentiated areas that later spread to differentiated areas, they transplanted these two regions, finding that only undifferentiated areas initiated new tumors. Decades later, Prince et al. (2007) fractionated human head and neck SCCs and identified a population comprising only 10% of the tumors but containing all their tumor-initiating cells, as evidenced by transplantation. Soon thereafter, the purification methods were refined to identify cells within skin SCCs, which at near single-cell levels generated SCCs when injected into host recipient mice (Schober and Fuchs 2011; Lapouge et al. 2012). These functional studies established the existence of bona fide SCC CSCs capable of efficiently initiating and propagating SCC tumors. Their purification also enabled their molecular characterization, unveiling new SCC CSC markers.

To identify tumor-initiating SCC CSCs within their endogenous tumor microenvironment, Blanpain and colleagues (Driessens et al. 2012) adapted the earlier radionucleotide tracing methods to genetic lineage tracing by using tamoxifen on *Krt14-CreER; Rosa26-fl-stop-flox-YFP* mice to monitor skin tumorigenesis. Like a tritiated thymidine pulse, this method marked proliferative progenitors of the tumors, enabling the researchers to identify a population of tumor cells that persisted long-term, self-renewed, and generated differentiated progeny.

Digging deeper, Fuchs and colleagues (Oshimori et al. 2015) exploited the fact that SCC CSCs are sensitive to transforming growth factor β (TGFβ), which had been reported to have both tumor-suppressing and tumor-promoting characteristics and to mark quiescent cancer cells (Malladi et al. 2016; Massagué and Sheppard 2023). To pinpoint when the tumor progenitors first experience a TGFβ signal, where it comes from, and what the consequences are, Fuchs and colleagues (Oshimori et al. 2015) designed a lentivirus harboring a TGFβ-sensitive enhancer element to drive both mCherry and a CreER and then coupled it to a tetracycline-inducible skin tumorigenesis system.

The initial TGFβ came from the monocytes associated with the emerging tumor perivasculature, which stimulated nearby tumor progenitors to express mCherry (Oshimori et al. 2015). By administering tamoxifen, these TGFβ-responding tumor cells could then be exclusively marked with YFP and lineage-traced, verifying their existence as bona fide cancer stem cells (CSCs), which first appeared at the benign, papilloma state and then expanded and contributed to cSCC progression (Yuan et al. 2022). Additionally, TGFβ-responding CSCs were slower cycling than their nonresponding counterparts but were invasive, showing signs of a partial epithelial-to-mesenchymal (EMT) transition at the SCC stage. The existence of SCC CSCs at invasive tumor edges is now well established (Oshimori et al. 2015; Chen et al. 2017; Ji et al. 2020).

TGFβ-responding CSCs hold promise as a therapeutic target. TGFβ signaling confers elevated chemoresistance to cSCC CSCs, and when it is abrogated, the cells succumb to cisplatin (Oshimori et al. 2015; Taniguchi et al. 2020). Interestingly, TGFβ production appears to arise from a feed-forward loop. TGFβ signaling increases NRF2-dependent production of interleukin 33 (Il-33), which interacts with its ST2 receptor on the surface of the nearby myeloid cells, prompting them to elevate TGFβ production and further stimulate the TGFβ-responsive cSCC CSCs (Taniguchi et al. 2020; Erickson et al. 2024). If these principles hold in humans as they do in mice and also in other SCCs, a new regimen for SCC treatment in the future might be to first kill the rapidly proliferating CSCs distal from a TGFβ signal, follow this with a TGFβ inhibitor to sensitize the slow-cycling CSCs, and then treat a second time with cisplatin to attack these newly mobilized CSCs, thereby preventing relapse. Other studies have also investigated targeting key downstream pathways of EMT expressed in CSCs together with chemotherapeutics to tackle these treatment-resistant cells (Debaugnies et al. 2023; Lengrand et al. 2023).

### SCC CSC transcriptional programs

Molecular exploration of SCC CSCs has been a critical avenue in the search to identify novel drugs that might target CSCs with minimal harm to their normal stem cell counterparts. Several enriched and/or unique gene expression programs have been identified in SCC CSCs, primarily from skin and head and neck in mice and humans (Beck et al. 2011; Schober and Fuchs 2011; Oshimori et al. 2015; Ge et al. 2016, 2017; Brown et al. 2017; Pascual et al. 2017; Pastushenko et al. 2021; Yuan et al. 2022; Taylor et al. 2024). These studies compared CSCs with their corresponding homeostatic stem cells or with unenriched basal progenitor cells. They identified a host of markers upregulated in CSCs that includes CD34 (Malanchi et al. 2008; Schober and Fuchs 2011), CD44 (Oshimori et al. 2015), SOX2 (Boumahdi et al. 2014), SOX9 (Ge et al. 2017), PITX1 (Sastre-Perona et al. 2019), CD200 (Stumpfova et al. 2010), VEGFA (Beck et al. 2011; Yuan et al. 2022), FAT1 (Latil et al. 2017; Pastushenko et al. 2018), and LEPR (Yuan et al. 2022) and pathways such as Tgfβ signaling and epithelial–mesenchymal transition (Oshimori et al. 2015; Taniguchi et al. 2020), angiogenesis (Beck et al. 2011; Yuan et al. 2022), lipid metabolism (Pascual et al. 2017), glutathione metabolism (Oshimori et al. 2015), and wound response (Ge et al. 2017).

Recent studies combining lineage tracing and extensive single-cell sequencing to construct gene correlation networks (“metagenes”) of mouse stem cells and their progeny as they progressed through benign and malignant states have identified that one cell population contains the CSC markers previously identified, thus strengthening the argument for a convergent CSC state (Taylor et al. 2024). This, along with many prior studies across various therapeutic regimens, suggest that it will be important to eliminate both proliferative and slower-cycling populations of the SCC CSCs (Oshimori et al. 2015; Brown et al. 2017; Taylor et al. 2024).

To understand the drivers of these unique SCC CSC gene expression programs, studies started delving into upstream mechanisms of these altered programs, including chromatin changes and transcription factor regulation. Some insights came from comparing the accessible chromatin profiles of cSCC CSCs with those of HFSCs and epidermal stem cells, which are considered the cells of origin of these cancers. When coupled with the histone modifications H3K27ac and H3K4me1 to mark the associated superenhancers (SEs), an SCC CSC chromatin landscape was revealed that was strikingly different from the normal tissue stem cells (Yang et al. 2015).

Motif analysis implicated several known (AP-1 and SOX2) and novel (KLF and ETS) SCC-associated transcription factors with further refinement in vivo with a core set of TFs (AP-1 and ETS) across all tumor cells that coordinates with the more epithelial-specific KLF and SOX2 motifs (Yang et al. 2015). Many of these changes were later confirmed in a second model (Latil et al. 2017). Knockdown of the SCC CSC ETS motif gene *Ets2* impaired tumorigenesis, whereas expression of a constitutively stabilized form of ETS2 in WT skin led to a cancer-like morphology accompanied by a chromatin landscape and gene expression program that bore striking resemblance to cSCC CSCs (Yang et al. 2015).

ETS2 activation is directly downstream from RAS/MAPK signaling, which causes ETS2 phosphorylation on threonine 72, enhancing its DNA binding and CBP/p300 interaction (Foulds et al. 2004). Although some of this gene expression program drives cell-intrinsic outcomes, ETS2 induces expression of chemokines CXCL1 and CXCL2, which can recruit neutrophils and induce angiogenesis (Yang et al. 2015). These findings present a model whereby induction of RAS/MAPK pathways leads to ETS2 recruitment to superenhancers in SCC CSCs, thereby triggering a gene expression program that drives further oncogenic behaviors by reprogramming both the CSCs and their tumor microenvironment.

## SCC CSC translational programs

The regulation of mRNA translation has emerged as a key driver in shaping cancer initiation, progression, and metastasis (Xu and Ruggero 2020; Fabbri et al. 2021; Kovalski et al. 2022). SCCs in particular face a harsh, resource-scarce environment that requires careful prioritization of oncogenic machinery over other pathways. Translational regulation plays a central role in this process; for example, via activation of the integrated stress response (Sendoel et al. 2017).

Cancer cells adeptly hijack the translational machinery—encompassing eukaryotic initiation factors (eIFs), ribosomal proteins, and noncoding RNAs (ncRNAs)—to devote limited resources toward synthesizing proteins most critical to fuel oncogenesis. In SCCs and other cancers, a central hub in translational control, eIF4E, is significantly elevated via both gene expression and activation by MAPK-mediated phosphorylation. Elevation of eIF4E also exhibits strong correlations with clinical outcomes and histological malignancy (Salehi et al. 2007; Liu et al. 2016; Dai et al. 2017; Srivastava et al. 2021; Tang et al. 2022). The augmented eIF4E activity in SCCs plays a role in pathogenesis by boosting the translation of key targets such as cyclin D1 and c-Myc, thereby promoting SCC proliferation and tumor growth (Srivastava et al. 2021).

The availability of transfer RNAs (tRNAs), the decoding components of the translation machinery, is an emerging mechanism by which SCCs develop as they exert regulatory control over translation. For instance, hypomethylation of tRNA at cytosine-5 (m^5^C), caused by the loss of NSUN2, triggers tRNA endonucleolytic cleavage and accumulation of 5′ tRNA fragments in cSCC. This results in global translation inhibition and increased self-renewal potential of tumor-initiating cells (Blanco et al. 2016). Additionally, tRNA N^7^-methylguanosine (m^7^G) modification by METTL1/WDR4 has been implicated in tumorigenesis through enhanced translation of oncogenic transcripts enriched in the MTOR signaling pathway and negatively regulating autophagy. This leads to m^7^G-related initiation and progression of esophageal and head and neck SCCs (Chen et al. 2022a; Han et al. 2022).

MicroRNAs (miRNAs) are small, well-conserved ncRNAs that can finely regulate gene expression by repressing translation or degrading RNA transcripts through binding to the 3′ untranslated region (UTR) of target mRNA in a sequence-dependent manner. Mounting evidence implicates aberrantly expressed miRNAs in cancer pathogenesis, including SCCs (Darido et al. 2011; Ma et al. 2011; Bhandari et al. 2013; Benaich et al. 2014; Zhang et al. 2014; Bracken et al. 2016; Ge et al. 2016). In SCCs, miR-21 functions in a feed-forward loop in which Grhl3 normally suppresses miR-21, but in Grhl3's absence, miR-21 is overexpressed, further reducing Grhl3, which is also a target of miR-21, resulting in loss of Grhl3 and its target, PTEN, and consequent PI3K/AKT/mTOR activation and tumorigenesis (Darido et al. 2011; Ma et al. 2011; Bhandari et al. 2013). Interestingly, miR-21(*) is also an oncomiR in SCCs via Rb/E2F (Ge et al. 2016). Besides miR-21/21(*), other miRs have been implicated in SCCs, such as miR-203, which inhibits metastasis independent of its effects on differentiation, and miR-125b, which promotes tumorigenesis via suppressing stress-responsive MAPK genes and indirectly prolonging EGFR signaling (Benaich et al. 2014; Zhang et al. 2014). Thus, microRNAs have protumorigenic or antitumorigenic effects in SCCs.

### Features of mRNA regulatory elements that drive translational regulation in SCCs

The translational machinery collaborates with mRNA *cis*-regulatory elements, including sequence motifs, modifications, and secondary structures, to selectively orchestrate protein synthesis (Blagden and Willis 2011; Schuster and Hsieh 2019). In cancer, this process is dysregulated in response to both internal oncogenic signals and external cues from the tumor microenvironment (Blagden and Willis 2011; Schuster and Hsieh 2019). Although our molecular understanding of translational dysregulation in cancer is just beginning to unfold, some tantalizing insights are emerging that underscore their importance in cancer progression.

One widely recognized *cis*-regulatory element for translation initiation is the upstream open reading frame (uORF) located in the 5′ UTR of mRNA. uORF usage enables the translation of oncogenic transcripts despite global translational suppression (Sendoel et al. 2017). The tumor environment often creates a stressful environment for CSCs, which encounter limiting nutrients, mechanical stress, and oxygen deprivation. This activates kinases that lead to the phosphorylation and incapacitation of the canonical translational initiator eIF2α. Upon activation of the integrated stress response, promiscuous noncanonical translational initiation can occur at CUGs and GUGs within certain 5′ UTRs of mRNAs, including those encoding MYC and CTNNB1 (Sendoel et al. 2017). Factors that have been implicated in oncogenic stress translation include EIF2A and EIF2B5 and an RNA helicase, DDX3, studied in cSCC and HNSCC, respectively (Sendoel et al. 2017; Chen et al. 2018; Cai et al. 2020). Although EIF2A and DDX3 promote SCC pathogenesis, EIF2B5 acts downstream from HRAS and oncogenic hyperproliferation to dampen self-renewal, perhaps to slow pathological growth in the face of oncogenic stress (Cai et al. 2020). Regulatory elements within 3′ UTRs are also used by SCC cells to selectively regulate translation and influence cancer development (Qie et al. 2017).

mRNA post-transcriptional modifications also play crucial roles in translational control underlying SCC pathogenesis. Notably, N^6^-methyladenosine (m^6^A), the most abundant RNA modification in mammalian cells, has been implicated in facilitating proliferation and metastasis in HNSCC (Liu et al. 2020; Chen et al. 2022b). Another prevalent modification is N^6^-methyl-2′-O-methyladenosine (m^6^Am), which is found at the first encoded nucleotide position adjacent to the m^7^G cap in 30%–40% of mRNAs. This modification has been found to influence mRNA stability, decapping, and translation during tumorigenesis (Wei et al. 1975; Grozhik and Jaffrey 2018). Phosphorylated RNA polymerase II CTD-interacting factor 1 (PCIF1) is the methyltransferase that collaborates with a binding protein cofactor to target specific mRNAs and catalyze m^6^Am modification of their m^7^G cap-proximal nucleotide. Recently, researchers discovered that PCIF1 is upregulated in head and neck SCC, where it targets and downregulates cap-dependent translation of the mRNA encoding a tumor suppressor and DNA demethylase TET2 to promote malignant progression (Li et al. 2023).

The important roles of translational regulation extend to cells within the tumor microenvironment of SCCs. A notable instance involves the response of cancer-associated fibroblasts (CAFs) to the proinflammatory cytokine IL-17, which is produced by immune cells in the tumor microenvironment and widely associated with poor outcome. In response to IL-17, CAFs orchestrate a collagen deposition program that effectively insulates the tumor from immune attack. Intriguingly, this program appears to be mediated by IL-17-induced translation of HIF1α—which is facilitated by direct binding of ACT1, the IL-17 receptor adaptor protein—to a stem–loop structure in the Hif1α mRNA 3′ UTR (Chen et al. 2022c). In this way, translational regulation within the tumor microenvironment may render SCCs refractory to anti-PD-L1 therapy (Chen et al. 2022c).

## Immune microenvironment of SCCs

Patients placed on immunosuppressive drugs for prolonged periods elevate their risk of developing cancers, especially nonmelanoma skin cancers and virally related tumors, pointing to the role of immunosurveillance of the surrounding tissue in preventing tumorigenesis. This is exemplified by the fact that organ transplant patients who are placed on immunosuppressive medications to prevent immune-mediated rejection are 65–250 times more likely to develop SCCs (Wysong 2023). This seems to be particularly true of cSCC, as other tumors even with high mutational burdens do not show the same degree of enhanced risk. The epithelium of the skin thus presents an example of how the immune system can simultaneously protect against environmental challenges and preserve tissue and organismal fitness. In the future, tapping into the mechanisms of T-cell-mediated surveillance and activation may be critical, as they can shape the T-cell repertoire even on distal tumors (Chen et al. 2023).

Despite immunosurveillance, as tumors develop, CSCs have devised mechanisms to evade cytotoxic T-cell destruction. To do so, they have acquired many signals to disarm the immune cells. One effective strategy across many tumors has been the PD1–PDL1 (programmed death ligand-1) pathway, which has limited ongoing cytotoxic T-cell activation. Indeed, most dramatically, in almost half of patients with advanced or metastatic cSCC, single-agent anti-PD1 therapy has been effective, presumably through reversing cytotoxic T-cell exhaustion within the tumor (Migden et al. 2018). In the SCCs of the lung, head and neck, esophagus, and cervix, cancer treatments can be quite effective when immunotherapy is paired with chemotherapy aimed at killing proliferative SCC cells. This multimodal therapeutic treatment has become the frontline treatment for metastatic disease, the efficacy of which may be due to the potential for targeting not only the proliferative cancer cells but also the more quiescent CSCs, providing that they express PDL1. Indeed, quiescent cancer cells evade detection even by antigen-specific T cells without PD-1 (Baldominos et al. 2022); therefore, these stem-like cells are the likely source of resistance to treatment with both chemotherapy and immunotherapy (Oshimori et al. 2015; Miao et al. 2019). Focusing on the immune-evasive strategies of these tumor cells may give us insight into new avenues for therapeutic development.

Whether mouse or human, SCCs from various body sites have been shown to express at least one of the B7 family checkpoint proteins, such as PDL1, CD80, and CD276, to dampen cytotoxic T-cell activity within the tumor microenvironment and/or delivered by adoptive T-cell transfer therapy (Xu et al. 2014a; Miao et al. 2019; Wang et al. 2021). Their expression is often seen at invasive fronts, where TGFβ signals to CSCs that reside at this transitory edge. TGFβ has been shown to trigger cSCC CSCs to express CD80 at sufficiently low levels to dampen rather than enhance a cytotoxic T-cell response (Miao et al. 2019). Such studies raise the possibility that additional ways to inhibit these checkpoints (or the upstream pathways that induce them) may be effective strategies for overcoming immune evasion if carefully regulated.

As more sequencing data across human cancers are being amassed, genetic changes that might facilitate immune evasion are also beginning to emerge, and with them come new challenges. One way is through loss of HLA class I or, alternatively, restriction of the neoantigens presented by the tumor. Recent studies show that lung SCCs, even compared with adenocarcinoma counterparts, have a high propensity to downregulate antigen-presenting machinery genes or key components of its structure, such as B2M (Li et al. 2015; Rosenthal et al. 2019). There is also a high rate of HLA class I loss of heterozygosity (LOH), which can limit T-cell-mediated destruction by preventing presentation of a diverse array of neoantigens on the tumor surface (McGranahan et al. 2017). Notably, downregulation of HLA class I has also been linked to poor outcomes in cervical SCCs, and HLA LOH is prevalent in esophageal SCCs (Mehta et al. 2008; Cui et al. 2023). These findings indicate that immune evasion through dampening of antigen presentation on the cancer cells may be a critical if not universal step in SCC pathogenesis. Therefore, methods to reverse this downregulation through epigenetic targets or to activate immune cells through non-MHC class I-dependent mechanisms may be effective strategies to explore in SCCs.

### Myeloid recruitment to the SCC microenvironment

Myeloid cells are abundant in almost all tumors, pushing out resident macrophages and acquiring distinct gene expression programs attuned to the tumor microenvironment (Lavin et al. 2017; Casanova-Acebes et al. 2021; Leader et al. 2021). Myeloid cells, including monocytes, macrophages, and neutrophils, can be seen as regulators of the tumor microenvironment: They play essential roles in fueling cancer progression through promoting angiogenesis, immunosuppression, and cancer metastasis (Xu et al. 2014a; Mollaoglu et al. 2018). During SCC-specific progression, monocytes are recruited to the tumor, where they differentiate into tumor-associated macrophages and alter the tumor microenvironment (Taniguchi et al. 2020).

SCCs actively recruit immature myeloid cells through secretion of monocyte-activating cytokines such as IL-33 (Taniguchi et al. 2020) or neutrophil-attracting chemokines such as CXCL5 (Mollaoglu et al. 2018) and result in accumulation of myeloid cells at the invasive edge, where they interact with the stem-like tumor cells. In the tumor microenvironment, these myeloid cells become more immunosuppressive as they differentiate to macrophages and elevate secretion of TGFβ, which in turn supports tumor-invasive CSCs in a feed-forward loop. Tumor-associated macrophages have also been shown to bar cytotoxic CD8^+^ T cells from the tumor by directly sequestering them and/or driving exhaustion (Chow et al. 2021; Kersten et al. 2022). Although epithelial cells harbor the potential for nonprofessional phagocytosis, recent studies suggest that macrophages may put selective pressure on tumor cells through phagocytic competition (Mesa et al. 2015; Ellis et al. 2019; Zhang et al. 2023). Understanding whether these features of macrophages play a role in heterogenous SCC tumors will be particularly fascinating, as tumor cell fitness helps determine the stark transition from noninvasive to invasive states. Although the direct role of macrophages in SCCs remains to be determined, their ability to produce and secrete active TGFβ confers at least an indirect role in cancer progression.

Other cell types within the tumor microenvironment, such as cancer-associated fibroblasts, also interact with cancer and immune cells and thus may influence treatment outcomes (Rinkevich et al. 2015; Foster et al. 2022). However, much more needs to be explored to elucidate the complex interplay between tumor cells and their surroundings, where little is known about the roles of innate lymphocytes, dendritic cells, different neuronal populations, and lymphatic capillaries, all of which function in epithelial tissues and are likely to play roles, positive or negative, in tumorigenesis and malignant invasion.

## Concluding remarks

**Figure 3.**
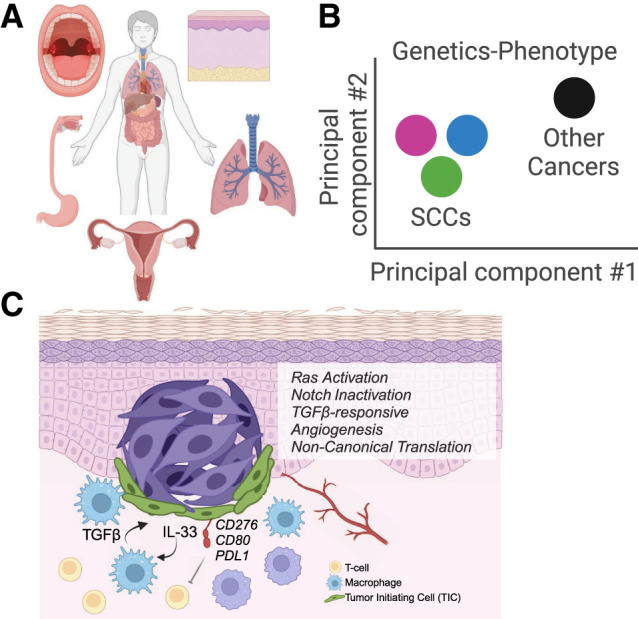
A unified perspective on SCCs. (*A*) SCC anatomical sites. Shown are (clockwise) head and neck (mouth), skin, lung, cervical, and esophagus. (*B*) Hypothetical PCA accounting for genetics and the molecular and clinical behaviors of SCCs versus other cancers. This illustrates how collectively SCCs are distinct from other cancers. (*C*) Squamous cell carcinoma engages multiple molecular and cellular pathways for initiation, progression, and therapeutic evasion, as described in the text. Created with BioRender.com.

Squamous cell carcinomas remain a major clinical challenge. Although they arise in distinct body sites, emerging evidence, presented here, points to shared biology, including genetics, cancer stem cell biology, gene expression programs and their regulation, signal transduction pathways, translational regulation, and interactions of SCCs with the microenvironment and immune cells (Fig. 3). Checkpoint inhibitors and RTK blockade have already had a major impact across SCCs, establishing the importance of understanding the molecular mechanisms underlying SCC malignancy and shared SCC pathways. Equally important in the quest for new therapeutics will be understanding more about the cross-talk between SCC CSCs and their tumor microenvironment, where CAFs, the vasculature, and the immune system play active roles in SCC tumorigenesis across various SCC body sites. Many of these cell types are starting to be explored in other contexts for novel cancer therapies. Although distinctions undoubtedly will arise, conceptualizing SCCs as one disease will be important in unifying current and past work and advancing novel therapies to help the many patients suffering from SCCs worldwide.
